# Association of Chemotherapy Timing in Pregnancy With Congenital Malformation

**DOI:** 10.1001/jamanetworkopen.2021.13180

**Published:** 2021-06-09

**Authors:** Mathilde van Gerwen, Charlotte Maggen, Elyce Cardonick, Emma J. Verwaaijen, Marry van den Heuvel-Eibrink, Roman G. Shmakov, Ingrid Boere, Mina M. Gziri, Petronella B. Ottevanger, Christianne A. R. Lok, Michael Halaska, Long Ting Shao, Ilana Struys, Elisabeth M. van Dijk-Lokkart, Kristel Van Calsteren, Robert Fruscio, Paolo Zola, Giovanna Scarfone, Frédéric Amant

**Affiliations:** 1 Center for Gynecological Oncology Amsterdam, Antoni van Leeuwenhoek–Netherlands Cancer Institute, Amsterdam, the Netherlands; 2 Princess Máxima Center for Pediatric Oncology, Utrecht, the Netherlands; 3 Department of Oncology, KU Leuven, Leuven, Belgium; 4 Department of Obstetrics and Gynecology, Cooper University Health Care, Camden, New Jersey; 5 National Medical Research Centre for Obstetrics, Gynaecology and Perinatology named after Academician V.I. Kulakov, Ministry of Healthcare of Russian Federation, Moscow, Russia; 6 Department of Medical Oncology, Erasmus MC Cancer Institute, Erasmus University Medical Center, Rotterdam, the Netherlands; 7 Department of Obstetrics, Cliniques Universitaires St Luc, Université Catholique de Louvain, Sint-Lambrechts-Woluwe, Belgium; 8 Department of Medical Oncology, Radboud University Nijmegen Medical Center, Nijmegen, the Netherlands; 9 Center for Gynecological Oncology Amsterdam, Amsterdam University Medical Centers, Amsterdam, the Netherlands; 10 University Hospital Kralovske Vinohrady, Third Medical Faculty, Charles University, Prague, Czech Republic; 11 Cooper Medical School, Rowan University, Camden, New Jersey; 12 Department of Child and Adolescent Psychiatry & Psychosocial Care, Emma Children’s Hospital, Amsterdam University Medical Centers, Amsterdam, the Netherlands; 13 Department of Obstetrics, University Hospitals, Leuven, Belgium; 14 Department of Development and Regeneration, KU Leuven, Leuven, Belgium; 15 Department of Obstetrics and Gynecology, San Gerardo Hospital, Milan, Italy; 16 Department of Surgical Sciences, University of Turin, Turin, Italy; 17 Gynecological Oncology Unit, Fondazione Istituto Di Ricovero e Cura a Carattere Scientifico, Ca’ Granda Ospedale Maggiore Policlinico Milan, Milan, Italy

## Abstract

**Importance:**

Chemotherapy during the first trimester of pregnancy should be avoided owing to the risk of congenital malformations. However, the precise gestational age at which chemotherapy can be initiated safely remains unclear.

**Objective:**

To assess congenital malformation rates associated with gestational age at initiation of chemotherapy among pregnant women with cancer.

**Design, Setting, and Participants:**

This multicenter cohort study evaluated all pregnant women who received chemotherapy between 1977 and 2019 registered in the International Network on Cancer, Infertility and Pregnancy (INCIP) database. Data were analyzed from February 15 to June 2, 2020.

**Exposures:**

Cancer treatment with chemotherapy during pregnancy.

**Main Outcomes and Measures:**

Analysis was focused on major and minor structural malformations in offspring, defined by EUROCAT, detected during pregnancy or at birth.

**Results:**

A total of 755 women in the INCIP database who underwent cancer treatment with chemotherapy during pregnancy were included in analysis. The median (range) age at cancer diagnosis was 33 (14-48) years. Among offspring, the major congenital malformation rate was 3.6% (95% CI, 2.4%-5.2%), and the minor congenital malformation rate was 1.9% (95% CI, 1.0%-3.1%). Chemotherapy exposure prior to 12 weeks gestational age was associated with a high rate of major congenital malformations, at 21.7% (95% CI, 7.5%-43.7%; odds ratio, 9.24 [95% CI, 3.13-27.30]). When chemotherapy was initiated after gestational age 12 weeks, the frequency of major congenital malformations was 3.0% (95% CI, 1.9%-4.6%), which was similar to the expected rates in the general population. Minor malformations were comparable when exposure occurred before or after gestational age 12 weeks (4.3% [95% CI, 0.1%-21.9%] vs 1.8% [95% CI, 1.0-3.0]; odds ratio, 3.13 [95% CI, 0.39-25.28]). Of 29 women who received chemotherapy prior to 12 weeks gestation, 17 (58.6%) were not aware of pregnancy, and 6 (20.7%) experienced a miscarriage (3 women [10.3%]) or decided to terminate their pregnancy (3 women [10.3%]).

**Conclusions and Relevance:**

This cohort study found that chemotherapy was associated with an increased risk of major congenital malformations only in the first 12 weeks of pregnancy. The risk of congenital malformations when chemotherapy was administered during the first trimester and the high number of incidental pregnancies during cancer treatment in the INCIP registry underscore the importance of contraceptive advice and pregnancy testing at the start of chemotherapeutic treatment in young women with cancer.

## Key Points

**Question:**

Is gestational age at initiation of chemotherapy during pregnancy associated with risk of congenital malformations?

**Findings:**

In this cohort study including 755 pregnant women with cancer, the risk of major congenital malformations was elevated when first chemotherapy exposure was prior to 12 weeks of gestation, whereas the occurrence of major congenital malformations was similar to expected rates in the general population when chemotherapy was initiated after 12 weeks of gestation.

**Meaning:**

The findings of this study could allow clinicians to better tailor chemotherapy during pregnancy and to inform patients on fetal risks of malformations.

## Teaser

This cohort study assesses the association of gestational age at initiation of chemotherapy among pregnant women with cancer with congenital malformation rates among their offspring.

## Introduction

Because chemotherapy attacks rapidly proliferating cells and is minimally selective, it also puts a developing fetus at risk of teratogenic effects. Toxic events during the periconceptional period might affect early embryogenesis and result in a miscarriage, whereas subsequent toxic exposure might interfere with the formation of organs, with the most susceptible period occurring between 2 and 8 weeks after conception (between 4 and 10 weeks postmenstruation).^1^ There is a wide consensus that chemotherapy should be administered until after organogenesis is completed, usually considered the first trimester of pregnancy (ie, the first 13 weeks postmenstruation).^1,2^ However, the exact timing of conception might be uncertain, and some systems (eg, eyes, genitals, hematopoietic system, central nervous system) continue to develop after 10 weeks of gestation. Therefore, the question remains in clinical practice: at what exact gestational age can chemotherapy be safely initiated to avoid inducing congenital malformations?

To assess the immediate teratogenic role of prenatal chemotherapy, this cohort study evaluated the presence of major and minor congenital malformations detected during pregnancy or at birth among the offspring of patients registered in the International Network of Cancer, Infertility and Pregnancy (INCIP).

## Methods

The Ethical Committee of Unity Hospitals of Leuven, Belgium, approved data collection for this cohort study. Prospectively registered patients provided written informed consent. Retrospectively registered paitents were deidentified, so the need for informed consent was waived. This study followed the Strengthening the Reporting of Observational Studies in Epidemiology (STROBE) reporting guideline for cohort studies.

The INCIP registry contains retrospectively and prospectively collected oncological, obstetric, and neonatal data, as well as offspring follow-up data of patients diagnosed with any pregnancy-associated malignant neoplasm, reported by physicians with a special interest in cancer in young women. Currently, there are 73 hospitals in 28 countries actively participating in INCIP. The registry was started in 2005, and this cohort study was performed with a data cutoff of December 1, 2019. Patient data, including gestational age at treatment initiation, duration of chemotherapy during pregnancy, and obstetric and neonatal outcomes, were collected for all pregnant women who received chemotherapy with known obstetric outcomes. Small for gestational age (SGA) was defined as a birthweight less than the 10th percentile, and percentiles were corrected for gestational age, sex, maternal height, maternal weight, ethnicity, and parity, according to the calculator from the Gestation Network (version 8.0.4; Perinatal Institute). Preterm delivery was defined as birth before 37 weeks gestational age. Congenital malformations were defined as structural or chromosomal malformations that were diagnosed prenatally or at birth. Classification in minor or major malformations was performed based on the medical, functional, or cosmetic consequences, according to EUROCAT guidelines.^3^

### Statistical Analysis

We used descriptive analyses and observed the numbers of reported congenital malformations according to gestational age at first chemotherapy exposure. The subgroup that initiated chemotherapy before 12 weeks of pregnancy was reported separately, and we defined the odds ratio (OR) and 95% CI for congenital malformations prior to 12 weeks of gestation. The χ^2^ test was used to compare occurrence of malformations between the group exposed to chemotherapy prior to 12 weeks and the group with exposure after 12 weeks. *P* values were 2-sided, and *P* < .05 was considered to indicate statistical significance for all analyses. Analyses were performed using SPSS Statistics version 25.0 (IBM). Data were analyzed from February 15 to June 2, 2020.

## Results

In total, 755 pregnant women treated with chemotherapy between 1977 and 2019 were included in analysis (Table 1). Median (range) maternal age at cancer diagnosis was 33 (14-48) years. Breast cancer was the most common cancer type (451 women [59.8%]), and most pregnancies ended in a live birth (745 women [99.4%]). A total of 27 neonates (3.6% [95% CI, 2.4%-5.2%]) were reported to have major congenital malformations, and 14 neonates (1.9% [95% CI, 1.0%-3.1%]) had minor congenital malformations. The occurrence of major congenital malformations was the highest if first chemotherapy exposure was prior to 12 weeks gestational age, at 21.7% (95% CI, 7.5%-43.7%), compared with 3.0% (95% CI, 1.9%-4.6%) congenital malformations among offspring of women who began chemotherapy after 12 weeks gestation (OR, 9.24 [95% CI, 3.13-27.30]), with the greatest risk for women who began chemotherapy periconceptionally (Figure and Table 2). The occurrence of major malformations when chemotherapy was initiated after 12 weeks of gestation was lower and remained stable with advanced pregnancy (Figure). The occurrence of minor malformations was comparable with the rates expected in the general population when exposure occurred prior or after 12 weeks gestational age (4.3% [95% CI, 0.1%-21.9%] vs 1.8% [95% CI, 1.0-3.0]; OR, 3.13 [95% CI, 0.39-25.28).

**Table 1.** Clinical Characteristics of Patients

Characteristics	Patients, No. (%)		
	Total (n = 755)	Gestational age at chemotherapy exposure	
		<12 wk (n = 29)	≥12 wk (n = 726)
Maternal age at cancer diagnosis, y			
Median (IQR) [range]	33 (30-36) [14-48]	32 (29-35) [19-41]	33 (30-36) [14-48]
<30	175 (23.2)	9 (31.0)	116 (22.9)
30-35	344 (45.6)	15 (51.7)	328 (45.2)
>35	236 (31.3)	5 (17.2)	231 (31.8)
Cancer type			
Breast	451 (59.7)	17 (58.6)	434 (59.8)
Cervical	59 (7.8)	0	59 (8.1)
Lymphoma	138 (18.3)	4 (13.8)	134 (18.5)
Leukemia	36 (4.8)	7 (24.1)	29 (4.0)
Ovarian	26 (3.4)	0	26 (3.6)
Gastrointestinal	27 (3.6)	0	27 (3.7)
Melanoma	1 (0.1)	0	1 (0.1)
Brain	3 (0.4)	1 (3.4)	2 (0.3)
Lung	4 (0.5)	0	4 (0.6)
Sarcoma	5 (0.7)	0	5 (0.7)
Other	5 (0.7)	0	5 (0.7)
Country of registration			
Belgium	146 (19.3)	4 (13.8)	142 (19.5)
Czech Republic	28 (3.7)	0	28 (3.9)
Germany	7 (0.9)	0	7 (1)
Denmark	9 (1.2)	1 (3.4)	8 (1.1)
Spain	5 (0.7)	0	5 (0.7)
Italy	82 (10.9)	0	82 (11.3)
Mexico	19 (2.5)	2 (6.9)	17 (2.3)
The Netherlands	162 (21.5)	9 (31.0)	153 (21.1)
Russia	67 (8.9)	3 (10.3)	64 (8.8)
United States	197 (26.1)	9 (31.0)	188 (25.9)
Israel	7 (0.9)	0	7 (1.0)
Other	26 (3.4)	1 (3.4)	25 (3.4)
Timing of cancer diagnosis			
Before pregnancy	14 (1.9)	7 (24.1)	7 (1.0)
First trimester	217 (28.7)	22 (75.9)	195 (26.9)
Second trimester	448 (59.3)	0	448 (61.7)
Third trimester	76 (10.1)	0	76 (10.5)
Pregnancy known at time of chemotherapy initiation			
No	23 (3.0)	17 (58.6)	6 (0.8)
Yes	732 (97.0)	12 (41.4)	720 (99.2)
Conception			
Spontaneous	689 (92.5)	25 (86.2)	673 (92.7)
ART	57 (7.5)	4 (13.8)	53 (7.3)
Prior pregnancies			
No	291 (38.5)	10 (34.5)	281 (38.7)
Yes	439 (58.1)	16 (55.2)	423 (58.3)
Not reported	25 (3.3)	3 (10.3)	22 (3.0)
Radiation therapy during pregnancy (first trimester)	18 (2.4)	1 (3.4)	17 (2.3)
Surgery during pregnancy (first trimester)	315 (41.7)	12 (41.4)	303 (41.7)
Chemotherapy regimen during pregnancy			
ABVD	73 (9.7)	1 (3.4)	72 (9.9)
Anthracyclines	320 (42.4)	5 (17.2)	315 (43.4)
Anthracyclines and taxanes	122 (16.2)	12 (41.4)	110 (15.2)
CHOP-like	53 (7.0)	3 (10.3)	50 (6.9)
Platinum-based	108 (14.3)	0	108 (14.9)
Methotrexate	1 (0.4)	1 (3.4)	0
Leukemia regimen	36 (4.8)	6 (20.7)	30 (4.1)
Temozolomide	3 (0.4)	1 (3.4)	2 (0.3)
Other	39 (5.2)	0	39 (5.4)
Gestational age at first chemotherapy exposure, wk			
Median (IQR) [range]	22.6 (17.9-27.1) [0-35.1]	8.5 (1.6-11.1) [0-11.7]	22.9 (18.4-27.3) [12.0-35.1]
0-3.9	10 (1.3)	10 (34.5)	0
4-9.9	9 (1.3)	9 (31.0)	0
10-13.9	42 (5.4)	10 (34.5)	32 (4.4)
14-27.9	535 (70.8)	0	535 (73.7)
28-40.0	159 (21.1)	0	159 (21.9)
Other medication during pregnancy			
G-CSF	77 (10.2)	8 (27.6)	69 (9.5)
Tamoxifen	2 (0.3)	2 (6.9)	0
Trastuzumab	3 (0.4)	0	3 (0.4)
Rituximab	41 (5.4)	0	41 (5.6)
Imatinib	1 (0.1)	0	1 (0.1)
GnRH analogue	1 (0.1)	1 (3.4)	0
Isotretinoine	1 (0.1)	1 (3.4)	0
Mercaptopurin	12 (1.6)	12 (41.4)	0
Pregnancy outcome			
Live birth	745 (98.7)	23 (79.3)	722 (99.4)
Stillbirth	4 (0.5)	0	4 (0.6)
Miscarriage	3 (0.4)	3 (10.3)	0
Termination	3 (0.4)	3 (10.3)	0
Singleton/multiple pregnancy			
Singleton	731 (96.8)	21 (72.4)	710 (97.8)
Multiple	18 (2.4)	2 (6.9)	16 (2.2)
Gestational age at delivery, wk (n = 745)			
Median (IQR) [range]	36.7 (34.9-38.1) [22.1-42.4]	37.3 (34.6-38.9) [29.4-40.6]	36.7 (34.9-38.0) [22.1-42.4]
<28	9 (1.2)	0	9 (1.2)
28.0-31.9	38 (5.1)	3 (13.0)	35 (4.8)
32.0-33.9	71 (9.5)	2 (8.7)	69 (9.6)
34.0-36.9	277 (37.2)	3 (13.0)	274 (38.0)
≥37.0	35(47.0)	15 (65.2)	335 (46.4)
Congenital malformations (n = 749)^a^			
None	708 (94.5)	17 (73.9)	691 (95.2)
Minor	14 (1.9)	1 (4.3)	13 (1.8)
Major	27 (3.6)	5 (21.7)	22 (3.0)

Abbreviations: ART, assisted reproductive technology; ABVD, doxorubicin, bleomycin, vinblastine, dacarbazine; CHOP, cyclophosphamide, doxorubicin, vincristine; G-CSF, granulocyte colony-stimulating factor; GnRH, gonadotropin-releasing hormone; IQR, interquartile range.

^a^ Comparison between occurrence of minor and major malformations between groups by Fisher exact: *P* < .01 for major malformations and *P* = .36 for minor malformations.

**Figure.**
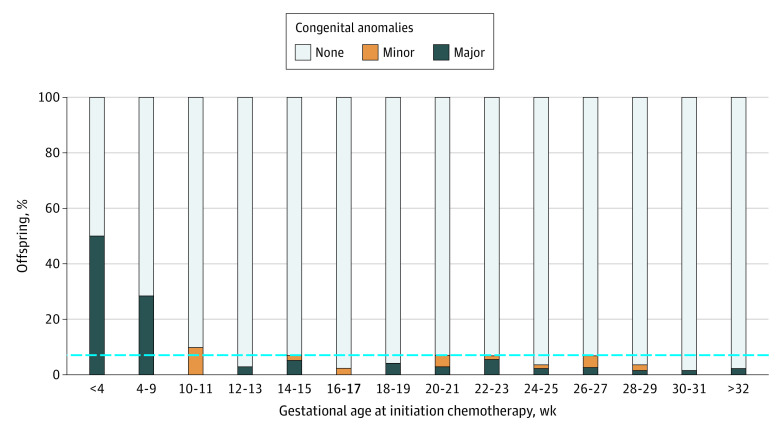
Frequency of Congenital Malformations According to Gestational Age at First Chemotherapy Exposure The dotted line indicates 7%, the maximum percentage of congenital malformations (minor and major) according to gestational age at initiation of chemotherapy, from 12 weeks onward.

**Table 2.** Overview of Pregnancy Outcomes in Women With Chemotherapy Exposure Prior to 12 Weeks of Gestation

Pregnancy outcome	No. (n = 29)	Chemotherapy regimen	Congenital malformation
**Initiation at <4 wk GA (n = 10)**			
Miscarriage	2	NR	NA
Termination	2	Polychemotherapy (n = 1) for AML	NA
		MTX (n = 1) for AML	
Live birth, no malformations	3	CHOP (n = 1)	NA
		Temozolomide (n = 1)	
		MTX (n = 1)	
Live birth, with malformations	3	Temozolomide (n = 1)	Microcephaly^a^
		TAC (G-CSF) (n = 1)	Limb abnormalities (bilateral III-IV syndactyly of hands and feet, and a hypoplasia of the right thumb)^a^
		TAC (tamoxifen + G-CSF + RT breast) (n = 1)	VSD and unilateral kidney agenesia^a^
**Initiation at 4-9 wk GA (n = 9)**			
Miscarriage	1	Polychemotherapy (n = 1) for AML	NA
Termination	1	Polychemotherapy (n = 1) for CLL	NA
Live birth, no malformations	5	Ara-C (n = 1)	NA
		FEC (n = 1)	
		AC (n = 3)	
Live birth, with malformations	2	CHOP (n = 1)	Epstein anomaly and dextrocardia^a^
		FEC (tamoxifen +GnRH agonist) (n = 1)	Limb abnormalities^a^
**Initiation at 10-11 wk GA (n = 10)**			
Miscarriage	0	NA	NA
Termination	0	NA	NA
Live birth, no malformations	9	ABVD (n = 1)	NA
		AC (n = 5)	
		AC (G-CSF) (n = 1)	
		EC (G-CSF) (n = 2)	
Live birth, with malformations	1	AC (n = 1)	Plagiocephaly

Abbreviations: ABVD, doxorubicin, bleomycin, vinblastine, dacarbazine; AC, doxorubicin, cyclophosphamide; AML, acute myeloid leukemia; Ara-C, cytarabine; CHOP, cyclophosphamide, doxorubicin, vincristine; CLL, chronic lymphocytic leukemia; EC, epirubicin; F, 5-flourouracil, cyclophosphamide; GA, gestational age; G-CSF, granulocyte colony-stimulating factor; GnRH, gonadotropin-releasing hormone; MTX, methotrexate; NA, not applicable; NR, not reported; RT, radiation therapy; TAC, doxorubicin, cyclophosphamide, docetaxel; VSD, ventricular sepal defect.

^a^ Major congenital malformation according to EUROCAT.

A total of 29 women initiated chemotherapy prior to 12 weeks of gestation. In 17 women (58.6%), pregnancy status was not known at the moment of chemotherapy initiation. A total of 6 patients (20.7%, all with hematological malignant neoplasms) experienced an early miscarriage after chemotherapy (3 women [10.3%]) or opted to terminate the pregnancy for oncological reasons (3 women [10.3%]). Of the remaining 23 neonates prenatally exposed to chemotherapy prior to 12 weeks of gestation, 6 (26.1%) had congenital malformations (Table 2). Notably, 2 children presented with very similar symmetrical limb deformations following exposure to anthracycline-based treatment (ie, docetaxel, doxorubicin; cyclophosphamide and 5-flourouracil, epirubicin, cyclophosphamide).

## Discussion

This cohort study presents the largest and most detailed cohort on congenital malformation occurrence according to gestational age at chemotherapy exposure, to our knowledge. We found an association between chemotherapy before 12 weeks of gestation and increased risk of congenital malformations detected during pregnancy or at birth. The overall congenital malformation rate among offspring of mothers who initiated chemotherapy after 12 weeks of gestation was 4.8%, which is comparable to the expected rates in the general population (ie, 2.5%-6.9% for major malformations and 6.5%-35.8% for minor malformations).^4,5,6^ Furthermore, 23 patients (3.0%) received chemotherapy without awareness of the pregnancy, underscoring the importance of adequate anticonception counseling and pregnancy testing at the start of chemotherapeutic treatment in young women with cancer.

To date, questions remain regarding when in the gestational period chemotherapy can be initiated relatively safely. First-trimester chemotherapy exposure has been associated with 10% to 20% risk of major malformations.^1^ Mechanisms by which chemotherapeutics induce teratogenic effects are incompletely understood. To date, the reported malformations after oncological treatment during human pregnancy encompass all organ systems, without discernible pattern for most cytotoxic drugs, except for aminopterin and methotrexate.^1^ The nature of teratogenesis is extremely complex; individual genetic susceptibility, specific timing of cytotoxic exposure, and specific type of (co-)medication all determine the spectrum of anomalies. Similar to this the findings reported in this study, other studies have reported limb deformities after exposure to a combination of cyclophosphamide and 5-fluorouracil in the first trimester of pregnancy.^7,8^ Most likely, this reflects chemotherapy-related toxic effects in the time frame when digits develop (ie, 5 to 6 weeks of gestation). However, proof of teratogenicity remains difficult because of other confounders, such as multidrug use, maternal age, and genetic predisposition.

We focused on structural malformations detected prenatally or at birth. Adverse effects and malformations can become apparent after birth as the eyes, genitalia, hematopoietic, and central nervous system continue to develop during childhood.^1^ Nevertheless, after birth, other confounders (eg, infections, medication use, environmental factors) play a role. A cohort study on 225 pregnant patients receiving chemotherapy after 12 weeks of pregnancy focused on structural birth anomalies diagnosed up to 5 years after birth and revealed an increased risk when chemotherapy was administered between 12 and 17 weeks of gestation.^9^ The causality of chemotherapy was unclear, as reported malformations were very heterogeneous (eg, pyloric stenosis, plagiocephaly, spina bifida) and could be also explained by other factors (eg, genetics, prematurity, folate deficiency).

Functional anomalies, sometimes subtle, might appear in early childhood or later. Among pediatric patients who were directly exposed to chemotherapy, anthracyclines are notorious for cardiotoxic effects, whereas platinum derivatives are associated with early ototoxicity.^10,11^ Another concern is the evolution of neurocognitive functions in the long term, since the central nervous system continues to develop during the second and third trimester of pregnancy. Postnatal exposure to chemotherapeutics has been associated with long-term genotoxic effects, such as a secondary malignant neoplasm and premature aging.^12,13^ To date, cohort studies on children exposed to chemotherapy prenatally report overall reassuring results, mostly based on general health, cardiac evaluation, and cognitive development until the age 6 years.^14^ Additionally, these clinical studies did not report on genotoxic effects after prenatal chemotherapy. Since the administration of chemotherapy in cancer treatment concerns combinatorial regimens of multiple chemotherapeutic agents and could differ per patient or hospital, reported results cannot provide information about the safety or risks of individual chemotherapeutic agents. Therefore, more research regarding genetic damage and developmental aspects with subsequent long-term follow-up is planned.

Data on the risks of congenital malformations are indispensable for clinicians and patients when considering chemotherapy during pregnancy. Based on our findings, we suggest that when cancer is diagnosed in early pregnancy, chemotherapy can be initiated from 12 weeks onward. Therefore, accurate ultrasonographic dating is crucial. The introduction of a 1-week safety period could be considered to further minimize the risk of chemotherapy-induced congenital malformations. However, no rationale exists to delay the start of chemotherapy beyond 14 weeks of gestation, as recommended previously.^2^ If a patient desires certainty on risk of chromosomal anomalies, an amniocentesis for karyotyping and microarray could be offered, since noninvasive prenatal testing is not conclusive in patients with cancer owing to tumor cell-free DNA interference.^15^

### Limitations

This study has some limitations. One important limitation of the INCIP cohort is that it is prone to selection bias, and data cannot be interpreted as population-based incidences. Based on this study, the absolute occurrence of anomalies after chemotherapy was impossible to assess, and minor anomalies are likely underreported. Additionally, early miscarriages and terminations of pregnancy are likely to be underrepresented in the INCIP registry, and with the substantial evolution of ultrasonographic imaging and improved prenatal diagnosis of congenital malformations over the years, more pregnancies might have been terminated. Furthermore, the use of multiple medications and treatment regimens in cancer treatment complicates the interpretation of results.

## Conclusions

These findings suggest that chemotherapy during the first 12 weeks of pregnancy was associated with increased risk for congenital malformations in the fetus. If an aggressive cancer diagnosis during early pregnancy does not allow treatment delay, parents should be counseled on fetal risks of malformations. If a patient incidentally becomes pregnant while receiving chemotherapy, prenatal counselling should include the risks of both short- and long-term adverse outcomes. Adequate anticonception and routine pregnancy tests should be offered to fertile women with cancer.

## Notes

Supplement. **Nonauthor Collaborators. **The International Network on Cancer, Infertility and Pregnancy
