# Supplementary material for: Association of Chemotherapy Timing in Pregnancy With Congenital Malformation
Source: JAMA Netw Open. 2021 Jun 9;4(6):e2113180. doi: 10.1001/jamanetworkopen.2021.13180 (PMC8190627; doi:10.1001/jamanetworkopen.2021.13180)
Supplement: Supplement. — Nonauthor Collaborators. The International Network on Cancer, Infertility and Pregnancy [file jamanetwopen-e2113180-s001.pdf]

Supplemental Online Content: Nonauthor Collaborators

*Indicates required information. Only first name, last name, and suffix will appear in PubMed.

*Group Name(s): International Network on Cancer, Infertility and Pregnancy (INCIP)							
***First Name and Middle Initial(s)**	***Last Name**	***Suffix (eg, Jr, III)**	Academic Degrees	Institution	Location (city, state/province, country)	Role or Contribution, eg, chair, principal investigator	Group (if more than 1 Group listed in the byline) and/or Subgroup (eg, Steering Committee)
Anthony	Richards		MD	The Royal Women's Hospital, Melbourne	Australia	Principal investigator	
Stephan	Polterauer		MD, PhD	Medical University of Vienna, Gynecological Cancer Unit, Vienna	Austria	Principal investigator	
Andrei	Pletnev		MD, PhD	N.N.Alexandrov National Cancer Center of Belarus, Dept. of Gynecological Oncology	Belarus	Principal investigator	
Sevilay	Altintas		MD, PhD	Antwerp University Hospital, Dept. Gynecological Oncology/ Breast Clinic	Belgium	Faculty participant	
Sileny	Han		MD, PhD	University Hospitals of Leuven, Dept. Gynecological Oncology	Belgium	Principal investigator	
Magali	Verheecke		MD, PhD	University Hospitals of Leuven, Dept. Gynecological Oncology	Belgium	Faculty participant	
Philippe	Tummers		MD, PhD	University Hospital Ghent, Women's Clinic	Belgium	Faculty participant	
Tessa	van Oostveldt		MD	AZ Middelares Gent, Dept. Gynecology and Obstetrics, Ghent	Belgium	Faculty participant	
Vincent	Rigo		MD	CHR Liège, Dept. Of Neonatology	Belgium	Faculty participant	
Eduardo	Paulino		MD	Brazilian National Cancer Institute, D	Brasil	Principal investigator	
Jaroslav	Klát		MD	University Hospital Ostrava, Dept. of Gynecological Oncology, Ostrava	Czech Republic	Faculty participant	

Supplemental Online Content: Nonauthor Collaborators

*Indicates required information. Only first name, last name, and suffix will appear in PubMed.

***First Name and Middle Initial(s)**	***Last Name**	***Suffix (eg, Jr, III)**	Academic Degrees	Institution	Location (city, state/province, country)	Role or Contribution, eg, chair, principal investigator	Group (if more than 1 Group listed in the byline) and/or Subgroup (eg, Steering Committee)
David	Cibula		MD, PhD	Dept. of Obstetrics and Gynaecology 3rd Medical Faculty, Charles University in Prague and Faculty Hospital Kralovske Vinohrady	Czech Republic	Faculty participant	
Kasper	Hjorth Ingerslev		MD	Odense Universitetshospital, Gynæk	Denmark	Principal investigator	
Lone	Storgaard		MD, PhD	Copenhagen University Hospital, Dept. of Obstetrics and Gynaecology	Denmark	Principal investigator	
Berit	Woetmann Pedersen		MD	Copenhagen University Hospital, Dept. of Obstetrics	Denmark	Faculty participant	
Cristel Maria	Sørensen-Hjortshøj		MD, PhD	Copenhagen University Hospital, Dep	Denmark	Faculty participant	
Mona	Aarenstrup Karlsen		MD, PhD	Copenhagen University Hospital, Dept. of Obstetrics and Gynaecology	Denmark	Faculty participant	
Paul	Berveiller		MD, PhD	Praticien Hospitalo-Universitaire, De	France	Principal investigator	
Dominik	Denschlag		MD, PhD	Hochtaunus Kliniken, Bad Homburg	Germany	Principal investigator	
Dimitrios	Haidopoulos		MD, PhD	Alexandra General Hospital, Dept. Gy	Greece	Faculty participant	
George-Marios	Makris		MD, PhD	Euroclinic Hospital, Dept. Gynaecolo	Greece	Faculty participant	
Ioannis	Kalogiannidis		MD, PhD	Hippokration Hospital, Aristotle Univ	Greece	Faculty participant	
Zoltan	Novak		MD	National Institute of Oncology, Dept.	Hungary	Principal investigator	
Lorenzo	Ceppi		MD	Ospedale San Gerardo, Clinica Ostetr	Italy	Faculty participant	
Monica	Fumagalli		MD, PhD	Fondazione IRCCS Ca' Granda Osped	Italy	Principal investigator	
Giorgia	Mangili		MD, PhD	San Raffaele Hospital Milan, Dept. of	Italy	Faculty participant	

Supplemental Online Content: Nonauthor Collaborators

*Indicates required information. Only first name, last name, and suffix will appear in PubMed.

*First Name and Middle Initial(s)	*Last Name	*Suffix (eg, Jr, III)	Academic Degrees	Institution	Location (city, state/province, country)	Role or Contribution, eg, chair, principal investigator	Group (if more than 1 Group listed in the byline) and/or Subgroup (eg, Steering Committee)
Fedro	Peccatori		MD, PhD	European Institute of Oncology, Fertility and Pregnancy in Oncology	Italy	Principal investigator	
Bianca	Masturzo		MD, PhD	Città della Salute e della Scienza di To	Italy	Faculty participant	
Anna	Fagotti		MD, PhD	Università Cattolica Roma	Italy	Faculty participant	
Matteo	Lambertini		MD, PhD	Policlinico San Martino-University of	Italy	Principal investigator	
Felice	Petraglia		MD, PhD	Azienda Ospedaliero Universitaria Ca	Italy	Faculty participant	
Davia	Vaitkiene		MD	Kaunas University of Medicine, Divisi	Lithuania	Principal investigator	
Adrius	Gaurilcikas		MD	Kaunas University of Medicine, Divisi	Lithuania	Faculty participant	
Alvaro	Cabrera Garcia		MD	Regional Hospital of High Specialty of	Mexico	Principal investigator	
Line	Bjorge		MD, PhD	Haukeland University Hospital, Dept.	Norway	Principal investigator	
Kristina	Lindemann		MD, PhD	Norwegian Radium Hospital, Oslo Un	Norway	Faculty participant	
Peter	Fedorcsak		MD, PhD	Oslo University Hospital, Dept. of rep	Norway	Faculty participant	
Hanne	Stennsheim		MD, PhD	Cancer Registry of Norway, Oslo	Norway	Faculty participant	
Kolawa	Wojciech		MD, PhD	Centrum Medyczne Macierzynstwo,	Poland	Faculty participant	
Kazimierz	Pitynski		MD, PhD	University Hospital of Krakow (Jagiell	Poland	Principal investigator	
Marta	Balajewicz-nowak		MD, PhD	University Hospital of Krakow (Jagiell	Poland	Faculty participant	
Stefan	Sajdak		MD, PhD	University Hospital of Krakow (Jagiell	Poland	Faculty participant	
Ewa	Kalinka		MD, PhD	Polish Mother's Memorial Hospital-R	Poland	Faculty participant	
Elzbieta	Lampka		MD	Maria Sklodowska-Curie Memorial Ca	Poland	Faculty participant	
Anna	Skrzypczyk-ostaszew		MD	Military Institute of Medicine, Warsa	Poland	Faculty participant	
Paula	Duarte		MD, PhD	Hospital de Vila Franca de Xira, Dept.	Portugal	Faculty participant	
Fátima	Cardoso		MD, PhD	Champalimaud Clinical Centre, Breas	Portugal	Faculty participant	
Tudor	Butureanu		MD, PhD	Elena Doamna University Hospital, D	Romania	Principal investigator	
Anastasia	Porokonnaya		MD	N.N. Blokchin National Medical Resea	Russia	Faculty participant	

Supplemental Online Content: Nonauthor Collaborators

*Indicates required information. Only first name, last name, and suffix will appear in PubMed.

***First Name and Middle Initial(s)**	***Last Name**	***Suffix (eg, Jr, III)**	Academic Degrees	Institution	Location (city, state/province, country)	Role or Contribution, eg, chair, principal investigator	Group (if more than 1 Group listed in the byline) and/or Subgroup (eg, Steering Committee)
Vladim	Bezrukkikh		MD	Almazov National Medical Research (	Russia	Faculty participant	
Elena	Ulrikh		MD, PhD	North-Western State Medical Univer	Russia	Faculty participant	
Igor	Govorov		MD, PhD	Almazov National Medical Research (	Russia	Faculty participant	
Alexey	Belyaey		MD	N.N. Petrov NMRC of Oncology, Saint	Russia	Faculty participant	
Sonsoles	Alonso Salvador		MD	Anderson Cancer Center, Dept. of Ob	Spain	Faculty participant	
Ignacio	Zapardiel		MD, PhD	La Paz University Hospital, Dept. Gyn	Spain	Principal investigator	
Blanca	Gil-Ibáñez		MD, PhD	University Hospital 12 Octubre, Dept	Spain	Faculty participant	
Sonia	Baulies		MD	Quiron-Dexeus, Barcelona	Spain	Faculty participant	
Natalia Rodriguez	Gómez-Hidalgo		MD	Vall D'hebron Hospital, Barcelona	Spain	Faculty participant	
Ernesto	Gonzalez-Mesa		MD, PhD	University Hospital Málaga	Spain	Faculty participant	
Elsa	Mendizábal		MD	University Hospital Gregorio Maraño	Spain	Faculty participant	
Kenny	Rodriguez-Wallberg		MD, PhD	Karolinska University Hospital, Dept.	Sweden	Principal investigator	
Chahin	Achtari		MD	Centre Hospitalier Universitaire Vauc	Switzerland	Faculty participant	
Christine	Brambs		MD, PhD	Luzerner Kantonsspital, Lucerne	Switzerland	Faculty participant	
Ruud	Bekkers		MD, PhD	Catharina Medisch Centrum, Dept. G	the Netherlands	Principal investigator	
Jorine	de Haan		MD, PhD	VU University Medical Center, Amste	the Netherlands	Faculty participant	
Christianne	de Groot		MD, PhD	VU University Medical Center, Dept.	the Netherlands	Principal investigator	
Sanne	Gordijn		MD, PhD	University Medical Center Groninger	the Netherlands	Principal investigator	
Judith	Kroep		MD, PhD	University Medical Center Leiden, De	the Netherlands	Principal investigator	
Rebecca	Painter		MD, PhD	Academic Medical Center Amsterdam	the Netherlands	Principal investigator	
Caroline	Schröder		MD, PhD	University Medical Center Groninger	the Netherlands	Principal investigator	
Teska	Schuurman		MD	Antoni van Leeuwenhoek, Dept. Gyn	the Netherlands	Faculty participant	
Ingeborg	Vriens		MD, PhD	Maastricht University Medical Cente	the Netherlands	Principal investigator	
Annemarie	Thijs		MD, PhD	Catharina Ziekenhuis, Dept. Medical	the Netherlands	Principal investigator	

Supplemental Online Content: Nonauthor Collaborators

*Indicates required information. Only first name, last name, and suffix will appear in PubMed.

***First Name and Middle Initial(s)**	***Last Name**	***Suffix (eg, Jr, III)**	Academic Degrees	Institution	Location (city, state/province, country)	Role or Contribution, eg, chair, principal investigator	Group (if more than 1 Group listed in the byline) and/or Subgroup (eg, Steering Committee)
Martine	van Grotel		MD, PhD	Prinses Máxima Center for pediatric	the Netherlands	Principal investigator	
Els	Witteveen		MD, PhD	University Medical Center Utrecht, D	the Netherlands	Principal investigator	
Anne	Armstrong		MD, PhD	The Christie NHS Foundation Trust, D	United Kingdom	Principal investigator	
Santiago	Scasso		MD, PhD	Pereira Rossell Hospital, Dept. Obste	Uruguay	Principal investigator	
Alexandra	Thomas		MD, PhD	Wake Forest Health Winston Salem,	USA	Faculty participant	
Clarissa	Bonanno		MD	North Shore University Hospital, Dep	USA	Faculty participant	
Jorge	Hoegl		MD	Servicio Oncologico Hospitalario (Uni	Venezuela	Principal investigator	
